# Service user and carer experiences of seeking help for a first episode of psychosis: a UK qualitative study

**DOI:** 10.1186/1471-244X-11-157

**Published:** 2011-09-30

**Authors:** Sanna Tanskanen, Nicola Morant, Mark Hinton, Brynmor Lloyd-Evans, Michelle Crosby, Helen Killaspy, Rosalind Raine, Stephen Pilling, Sonia Johnson

**Affiliations:** 1 Early Intervention Service, Camden and Islington NHS Foundation Trust, 4 Greenland Road, London, NW1 0AS, UK; 2 Department of Social and Developmental Psychology, University of Cambridge, Free School Lane, Cambridge CB2 3QR, UK; 3 Department of Mental Health Sciences, University College London, Charles Bell House, 67-73 Riding House Street, London, W1W 7EJ, UK; 4 Department of Epidemiology and Public Health, University College London, 1-19 Torrington Place, London, WC1E 7HB, UK; 5 Centre for Outcomes Research and Effectiveness, Division of Psychology and Language Sciences, University College London, 1-19 Torrington Place, London, WC1E 7HB, UK

## Abstract

**Background:**

Long duration of untreated psychosis (DUP) is associated with poor outcomes and low quality of life at first contact with mental health services. However, long DUP is common. In order to inform initiatives to reduce DUP, we investigated service users' and carers' experiences of the onset of psychosis and help-seeking in two multicultural, inner London boroughs and the roles of participants' social networks in their pathways to care.

**Method:**

In-depth interviews were conducted with service users and carers from an early intervention service in North London, purposively sampled to achieve diversity in sociodemographic characteristics and DUP and to include service users in contact with community organisations during illness onset. Interviews covered respondents' understanding of and reaction to the onset of psychosis, their help-seeking attempts and the reactions of social networks and health services. Thematic analysis of interview transcripts was conducted.

**Results:**

Multiple barriers to prompt treatment included not attributing problems to psychosis, worries about the stigma of mental illness and service contact, not knowing where to get help and unhelpful service responses. Help was often not sought until crisis point, despite considerable prior distress. The person experiencing symptoms was often the last to recognise them as mental illness. In an urban UK setting, where involved, workers in non-health community organisations were frequently willing to assist help-seeking but often lacked skills, time or knowledge to do so.

**Conclusion:**

Even modest periods of untreated psychosis cause distress and disruption to individuals and their families. Early intervention services should prioritise early detection. Initiatives aimed at reducing DUP may succeed not by promoting swift service response alone, but also by targeting delays in initial help-seeking. Our study suggests that strategies for doing this may include addressing the stigma associated with psychosis and community education regarding symptoms and services, targeting not only young people developing illness but also a range of people in their networks, including staff in educational and community organisations. Initiatives to enhance the effective involvement of staff in community organisations working with young people in promoting help-seeking merit research.

## Background

The onset of psychosis is a significant, sometimes catastrophic health event for individuals and their carers. With onset typically in late adolescence and early adulthood, if psychotic illness advances without intervention, the likelihood of treatment resistant symptoms, permanent psychosocial delay and a life-time reliance on health and social systems increases [1,2]. Long duration of untreated psychosis (DUP) independently predicts poor outcomes [3,4] and is associated with poor quality of life at first contact with mental health services [3]. However, lengthy DUP is common: a recent systematic review found mean DUP of over two years [3]; studies have reported median DUP of over 6 months in standard services [5,6]. In 2001 the UK Department of Health committed itself to funding early intervention services [7] with the aim of improving prognosis through intensive treatment delivered at the earliest point following onset of a first psychotic episode and maintained through an initial 3-5 year 'critical period' [8,9]. Fifty Early Intervention Services commissioned across the UK were tasked with developing and implementing an early detection strategy [7]. In line with this policy, reduction in duration of untreated psychosis (DUP) is a key performance indicator by which the effectiveness of UK early intervention services is judged.

Many factors may contribute to the typically long treatment delays for people experiencing a first episode of psychosis. These include poor individual, familial and community education about the signs and symptoms of psychosis, reluctance to accept stigma-laden diagnoses and the pervasive mistrust of mental health services within the general community [10-13]. High thresholds for inclusion amongst overly-stretched services, apathetic rather than curious health professionals and poor intra and inter organisational communication have also been laid to blame [14-16].

A recent systematic review of initiatives to shorten DUP suggested that successful early detection initiatives promoted prompt help-seeking in addition to minimising health service delays once help had been sought [17]. A quantitative study of pathways to care in Birmingham UK [18] found substantial delays both in initiating help-seeking and in health service responses for a first episode psychosis sample. Low rates of attendance and problems in communications with GPs have been found for young people in general [19]. An audit of pathways to care for people with first onset psychosis in inner London [20] found that only a minority of young people were registered with a GP or other health agency at the time of illness onset. A need to involve people experiencing psychosis, their families or people working in non-health organisations more directly in the help-seeking process is therefore indicated. North American research has found that non-health professionals are commonly involved in pathways to care for people with a first onset of psychosis [21] and that pathways involving non-medical professionals were associated with longer DUP [22]. This suggests non-health service community organisations and professionals could be a target for early detection interventions. However, there is little research on the experiences of help-seeking within the UK healthcare and social system of people with first episode psychosis and their families.

## Aim

We investigated service users' and carers' experiences of the onset of psychosis and help-seeking in two inner-city London boroughs. A particular focus was the roles of relevant community groups and non-health professionals in pathways to care, a relatively unexplored area to date within the early detection literature. A qualitative approach was used to gather in-depth accounts of initial help-seeking processes, with the aim of informing and improving the effectiveness of a local early detection strategy. In our analysis, we aimed to identify potential routes to earlier mental health service contact following the onset of psychosis.

## Methods

### Setting

The research took place in the London boroughs of Camden and Islington, which are socially and ethnically diverse and include areas of high deprivation. Participants were drawn from Camden & Islington NHS Foundation Trust Early Intervention Service (CIEIS), which offers intensive treatment for up to three years to people aged 18 to 35 with a first episode of affective or non-affective psychosis.

### Participants

Our sample of CIEIS service users and carers (not matched to service user respondents) was purposively recruited: a) we prioritised service users who were in contact with community organisations at the time of referral to CIEIS, in order to explore the potential role of community groups (such as education, housing, employment and young people's services and local faith and cultural organisations) in help-seeking; b) we sought diverse participants in terms of age, gender, ethnic group, educational attainment, employment history and duration of untreated psychosis. Participants were required to understand and speak adequate levels of English and be able to give written informed consent.

### Measures

Topic guides for semi-structured interviews with service users and carers covered: onset of difficulties; main activities and contact with community organisations at the time mental health problems developed; respondents' understanding of and responses to symptoms; help-seeking attempts; reactions from social network to the onset of illness; and experiences of help-seeking and service responses. Additional probes were used to elicit more information as appropriate.

### Procedures

Participants were recruited via CIEIS clinical staff. Interviews lasted an hour on average and took place at CIEIS or respondents' homes. They were conducted by MC and MH.

### Analysis

Interview transcripts were entered into QSR NVivo7 qualitative analysis software and analysed using thematic analysis [23]. We used both deductive and inductive approaches, seeking answers to our initial research questions whilst also exploring themes that emerged directly from the data [24]. The analytic process involved the development of a thematic framework to capture recurrent and underlying themes through a cyclical process of reading, coding, exploring the patterning and content of coded data, reflection and team discussion. Analysis was conducted by ST with N.M. and B.L.E. providing additional input regarding checking coding of transcripts and development of the coding frame to enhance validity. This collaborative approach resulted in a hierarchical thematic framework in which higher order themes represented more general or over-arching topics or issues and sub-themes reflected variations, reasons for or sub-components of these. This informed the structure and contents of our results with a primary focus on help-seeking experiences and impediments. Interview extracts that capture the main themes and experiences expressed are provided.

## Results

### Participant characteristics

21 service users and 9 carers were interviewed for the study. Service user participants' characteristics and duration of DUP are shown and compared to those for a representative sample of CIEIS clients in Table 1. Carer participants' characteristics are shown in Table 2.

**Table 1:** Demographic Characteristics of the Service User Sample (N = 21) and a CIEIS comparison

Characteristics	Category	Respondents n (%)	Overall CIEIS (%)*
Gender	Female	6 (28.6%)	40%
	Male	15 (71.4%)	60%
Age	Mean age	26.5 (SD 5.07)	23.5 (SD 5.57)
Ethnicity	White British	3 (14.3%)	41.1%
	White Other	4 (19%)	14.2%
	Black African	3 (14.3%)	14.2%
	Black Caribbean	5 (23.8%)	5.8%
	Asian Bangladeshi	4 (19%)	7.4%
	Mixed Race	2 (9.5%)	7.5%
DUP	Median DUP (days)	106	118
	< 3 months	10/21 (48%)	48/117 (41%)

SD = Standard deviation.

* Figures are based on overall CIEIS service user statistics in 2009.

**Table 2:** Demographic Characteristics of the Carer Sample (n = 9)

Characteristics	Category	Frequency
Gender	Male	1 (11.1%)
	Female	8 (88.9%)
Age	26-33	2 (22.2%)
	49-59	5 (55.6%)
	60-68	2 (22.2%)
Ethnicity	White British	5 (55.6%)
	White Other	2 (22.2%)
	Black Caribbean	1 (11.1%)
	Mixed Race	1 (11.1%)
Relationship	Mother	6 (66.7%)
	Sister	1 (11.1%)
	Partner	1 (11.1%)
	Mother-in-law	1 (11.1%)

The study sample included a higher proportion of service users from non-white ethnic groups than the CIEIS clinical population as a whole. Typical DUP and the proportion of service users with short DUP (< 3 months) were broadly representative of all CIEIS service users. All but one of the carer sample were female.

### Findings

Multiple sources of treatment delay were found, attributable to individuals, their social networks and services. The themes and experiences reported by service users and carers were generally congruent: we report differences in perspective where they were found. Results are organised thematically, describing participants' responses to the development of symptoms, reactions from their social networks and experiences of contact with services. Quotations illustrating major themes are presented in the text; additional illustrative quotations are provided in Additional File 1.

### 1) Understandings of symptoms and experiences

#### Attribution of symptoms

The majority of service user participants (n = 18) described a period (varying from a few weeks to years) in which they had not understood their experiences as being a form of mental health problem or something for which help from health services might be available.

*"I just thought they [symptoms] were normal, I thought everyone got them. Obviously everyone didn't get them." (Service user; male, 20, White British) *(see also Additional File 1, 1.1)

Carers reported similar difficulties in recognising service users' problems as signs of psychosis, and for many (n = 6), this was associated with retrospective feelings of frustration or guilt for not having recognised symptoms earlier and thus potentially prolonging the suffering of their family members. These delays were attributed to the vagueness of early symptoms and to lack of awareness of psychosis at individual and community levels.

*"[I] didn't have a clue. There is no history of anything like that in my family so we had no experience of it whatsoever.[...]No, I didn't know what it was and I was really frightened." (Carer; mother, 58, White Other) *(see also Additional File 1, 1.2)

Service users reported alternative explanations for psychotic symptoms including substance misuse, stress, physical illness, depression, sleep deprivation and religious experiences. Carers cited rebellious teenage behaviour, illicit drug use, stress, physical and neurological conditions, other psychological problems such as post-natal depression or personality characteristics.

"At the time I really didn't know what was happening and I only felt I was under a lot of stress, I didn't feel that I was going to have psychosis." (Service user; male, 26, Asian Bangladeshi)

*"If I talked to people they would say 'he sounds like a normal teenager to me'. You do sort of wonder, because the other two had not been like this at all, you wonder whether if this is what they mean by 'stroppy obnoxious teenagers' and so you put it down to that". (Carer; mother, 49, White British) *(see also Additional File 1, 1.3)

#### Response to symptoms

Almost half the service users (n = 10) and one third of the carers (n = 3) described thinking symptoms were transient and would resolve without the need for further intervention. These accounts seemed to be linked to longer duration of untreated psychosis and attribution of symptoms to other causes such as developmental phase.

*"Well, for the first week that I was hearing them [voices], I thought if I just stayed in my room and went to sleep it would, I'd just wake and it would stop, but it didn't." (Service user; female, 27, Black Caribbean) *(see also Additional File 1, 1.4 and 1.5)

Many service users (n = 13) described withdrawing from their social networks as a response to their symptoms.

*"I was starting to get a bit more, like, enclosed, like I didn't want to like socialise with people. I felt as if everyone out there was out to get me or something like that, like I just didn't want to like, talk to anyone. I felt moody I felt as if everybody was just invading my space or I was invading theirs." (Service user; female, 25, Asian Bangladeshi) *(see also Additional File 1, 1.6)

Some service users (n = 8) reported actively disguising psychotic symptoms from others, through a desire to preserve their self-image and appear normal to others

*"He [father] knew for a long time. He told me you seem really unhappy. Now he says 'you seemed really unhappy I knew something was wrong'. But because I wouldn't speak to him or open up I would just say 'that's fine, its fine'. I would try and avoid him rather than talk to him. He couldn't get anything out of me." (Service user; male, 21, White British) *(see also Additional File 1, 1.7).

### 2) Help-seeking processes

Service users and carers describe change over time and ambivalence in their response to difficulties. While many service users shifted between temporarily acknowledging a need for help and denial of or alternative explanations for their difficulties, three main responses were reported:

#### a) Unawareness of problems

Eleven respondents describe remaining unaware of their psychosis until contact with mental health services. Help-seeking for these individuals was therefore often complicated, prolonged and involved various attempts to intervene by family and friends, community organizations, statutory and emergency services. In some cases, help-seeking was initiated without service users' knowledge and/or consent.

*"I went to get a sick note from the GP and I explained some of the experiences that I'd had, which for me was of no concern at all, it was perfectly normal a lot of the things that had happened, but I needed time to kind of you know process the things. But for the GP it sounded like 'Oh my God', you know' [...]. So I was then referred onto this other place over here [mental health service]." (Service user; female, 34, White Other) *(see also Additional File 1, 2.1).

#### b) Attribution of problems to mental illness

Six service users reported gradually acknowledging their problems as mental ill-health and subsequently initiating help-seeking. These respondents appeared to recognise a need for help as their symptoms became unmanageable and culminated in crisis. Consistent encouragement and pressure from others to seek help aided help-seeking.

"I did wait a few days 'cause I was scared, but then the voices started to tell me to cut my throat and I nearly did. So I got scared and I went to the doctors." (Service user; female, 27, Black Caribbean)

"About two years ago, nearly three years ago, things like started to pop up in my head whereas before I used to think about it, the things that were talking to me and that's when I thought there is something definitely going on because like someone actually talking to me in my head isn't right." (Service user: female, 25, Asian Bangladeshi)

#### c) Other attributions of problems

The remaining 4 service users acknowledged a need for help but did not view their difficulties as mental health problems, so instigated more general help-seeking.

"I thought okay there is something wrong with me. Then I kept phoning the ambulance because I thought I was having a heart attack and it was really weird." (Service user; male, 21, White Other)

In contrast, all the carers came to recognize a need for help although the time-frame for help-seeking varied considerably. Carers noticed uncharacteristic and bizarre behaviours which alerted them to consider taking action, although a majority (n = 5) reported that help-seeking was not initiated until a crisis point was reached.

*"[...] My house was full of relatives so I wasn't completely focused on her but I was noticing she was behaving oddly, she was kind of disengaged. She was saying odd things, she was talking inappropriately to the children, like 'Don't listen to your mummy' or 'Don't do this' totally odd and not Mary at all [...] She was just completely spaced out. I took her to the doctor right that minute, early evening, because it was a build up over that week, over a couple of days, but it was very quick. She did go into it very quickly." (Carer; mother, 58, White Irish) *(see also Additional File 1, 2.2 and 2.3)

Many carers (n = 8) discussed the service user's lack of acknowledgement of their psychosis and reluctance to get help as a barrier for contacting services. All the carers described having tried to convince their family member to seek help but often faced denial, anger and stigma-related worries that consequently delayed appropriate help-seeking.

"I don't think he wanted any help. He wasn't really acknowledging that he had a mental illness. [...] when you tried to talk to him he became very defensive." (Carer; mother, 59, White British)

### 3) Beliefs and knowledge about mental health services

#### Stigma

Most service user respondents (n = 15) reported concerns about stigma as a barrier to help-seeking: fear of negative reactions to mental illness from others (n = 12); fears about mental health services (n = 5); and fears about the social consequences of mental health service involvement (n = 5).

"They were like 'go to the doctors and tell them what's wrong'. Because I didn't know about the illness I used to say like 'no because then they will lock me up, they will think I am crazy and stuff'." (Service user; male, 20, White British)

*"I was worried about they might think I was mental and take me away from my family and things like that. I don't know... mess me up." (Service user; male, 19, Asian Bangladeshi) *(See also Additional File 1, 3.1)

Carers were predominantly concerned about the potential adverse social and psychological consequences of their family members entering the mental health system. They also expressed worries about treatment and misgivings about mental health services.

*"They [relatives] were all saying the same thing, he needed to go to services, but underlying that there were issues that should he really go to services because they are just going to pump him up with drugs, give him an injection and section *[compulsorily detain] *him off and we'd never see the John that we know and love again. So that was a huge concern for everybody including myself." (Carer, mother, 51, Black Caribbean) *(see also Additional File 1, 3.2).

Similar concerns were expressed across all ethnic groups within the service user sample. Only one person (of Black African origin) reported a concern about contact with mental health services relating directly to their ethnic background.

#### Lack of knowledge

Identifying an appropriate service and route to treatment had been difficult for both service users and carers. Over half of service users (n = 12) talked about not having adequate knowledge about mental health services and the types of help available at illness onset. Six reported thinking that help did not exist for the psychotic symptoms they were experiencing.

*"I didn't even know that the services existed for these problems. I really was totally oblivious to mental health." (Service user; male, 28, Mixed Race) *(See also Additional File 1, 3.3)

Similarly, most carers (n = 6) reported having insufficient knowledge about mental health services at this time and feeling uncertain about where to seek help. The vagueness of symptoms added to their feelings of confusion and made it harder to determine the type of help needed.

*"I suppose not knowing what to do is always one of the hardest things. Because even taking them to the doctor is a very vague thing. There is nothing you can put your finger on and say. [...] Because it's such a vague thing it's hard to know where to go and who to talk to." (Carer; mother, 49, White British) *(See also Additional File 1, 3.4)

### 4) Responses of social networks to illness onset and help-seeking

#### Responses of immediate social networks

Although most service users (n = 19) reported being encouraged to seek help by their immediate social networks (friends, family members and partners), nine had also experienced unhelpful responses ranging from not recognizing or minimising the severity of illness to alarming or critical responses. One third of carers also described unhelpful responses from others in their social network when they had tried to instigate help-seeking. These had delayed or discouraged contact with mental health services.

*"My girlfriend at the time had been carrying all these leaflets about bipolar disorder and stuff like that erm and actually she didn't help in the slightest, she was like 'they will probably section *[compulsorily detain] *you if they diagnose you with a mental health issue'." (Service user; male, 24, White British)*

*"My mother would often get very irritated and say 'what do you want us to do - put her in a mental asylum?' like that was the most horrible thing one could say. [...] My father used to say 'oh let her go out into the world, she'll soon be put in her place or something...'" (Carer; sister, 33, White British) *(see also Additional File 1, 4.1).

#### Responses of community organisations

Prior to their first contact with mental health services, 17 service users described being in contact with one or more community organisation. These included religious organisations, employment services, universities and colleges and youth and leisure groups.

Nine respondents reported that their difficulties had initially gone unnoticed or were ignored by community group staff.

*"I was talking to myself a lot and I was having...and I was laughing to myself and smiling to myself and joking to myself and he *[my priest] *sort of realised that there was something not right. But he used to try and avoid me which wasn't very good." (Service user; female, 37, Black Caribbean) *(see also Additional File 1, 4.2 and 4.3)

Service users had been reluctant to approach community staff. Six of the eight who were in education said that they had not thought to approach their tutors for help, as they perceived these relationships to be distant, impersonal and inappropriate for discussing mental health difficulties. Similar concerns were described by two others about employers.

"I wasn't really sure who to go to, to be honest with you. When you are at university it feels like people are more occupied with the marks you are getting than how you are doing mentally. It's difficult to cope sometimes and it's difficult to know who to talk to and ask for help." (Service user; female, 25, Mixed Race)

*"Because I think mental health in a working environment or any kind of medical issue in a working environment that has to do with your head, they would rather get rid of you, rather than tell you to go and get help." (Service user; male, 26, Mixed Race) *(see also Additional File 1, 4.4)

Positive responses from community organisations were also reported. For six service users, encouragement from non-health community organisations led to referrals to mental health services either directly or via a GP. The involved workers were the respective participants' employer, son's nursery teacher, hostel support worker, university counsellor, youth worker and prison officer. Three more service users described receiving advice from community staff to seek help but not acting on it. Initial contact with mental health services was eventually instigated in these cases by family members or emergency services.

*"And he *[employer] *suggested I talk to my priest, but it was just like, at that time I suspect I was a little bit beyond reach of anyone who didn't really know what they're doing." (Service user; male, 24, White British)*

Seven carers discussed their family member having been in regular contact with occupational, educational or religious community groups prior to contact with mental health services. In three cases, schools and occupational health had noticed a deterioration of functioning but this had not led to a referral to mental health services. In four cases the community organizations had not instigated help-seeking or raised any concerns. However, two carers whose family members were in contact with church youth groups perceived their support to have been valuable, even though they had not advocated help-seeking.

*"Their [church] response was they were praying for him and encouraged him to come. Just spoke to him. There is a youth group within the church and I think really that was his saving grace, that gave him insight. Because when he got sectioned *[compulsorily detained] *after that he continued to go to the church and today he is a strong man of the Lord. So I really do believe that that has pulled him through." (Carer; mother, 51, Black Caribbean).*

### 5.) Health professionals' responses

Although for most respondents, help-seeking was initiated by a member of their social network, several respondents (n = 6) themselves contacted health professionals, who did not then initially recognise psychosis. Some respondents were reluctant to divulge problems and correspondingly vague when describing symptoms, often emphasizing physical problems over mental health difficulties.

"Went into the GP and the GP didn't really help. He said okay let's give it time and see. He thinks that was still with the physical symptoms. Just physical symptoms. So the GP didn't do much. So when the symptoms got worse then I actually got admitted to hospital and then things started rolling." (Service user; male, 27, White Other)

*"I told him [GP] that I was having burning feelings and something was wrong with my heart and that when that happened I was like 'huh, huh' I couldn't breathe at all. I was really messed up and she thought I had asthma or something and they gave me some asthma pumps. I tried it but it didn't really help I just had to wait for it to wear off. It took a long time and I was really messed up." (Service user; male, 19, Asian Bangladeshi) *(see also Additional File 1, 5.1).

Seven carers reported receiving what they viewed as uninformed or insensitive responses from health and educational professionals.

"So I brought him along to our local GP and told her what I felt was really wrong with him and she kind of dismissed it and said 'no, I think he just needs to sleep, he needs some sleeping tablets and he needs to sleep'. [...] She [GP] said 'No, no give him these tablets and he'll be fine'. And his situation got worse." (Carer; mother, 51, Black Caribbean)

*"He had a week or two off, I went in and saw the staff [sixth form college], I went in with him and said that he was having difficulties. They didn't take it on board." (Carer; mother, 60, White British) *(see also Additional File 1, 5.2 and 5,3).

In four cases, carers considered that detection of psychosis had been further delayed by their family member not disclosing positive symptoms to family or health care professionals (see Additional File 1, 5.4).

## Discussion

### Main Findings

Our study confirms that multiple, complex factors contribute to treatment delay for first episode psychosis, including not attributing problems to mental illness, stigma-related concerns, lack of knowledge about where to go for help and unhelpful health service responses. Our results concur with previous studies [16,25] in suggesting that a crisis point or overtly socially unacceptable behaviour is often the catalyst to seeking help, despite considerable prior distress for both the individual experiencing symptoms and their family. Mixed findings from previous research regarding whether family members' involvement promotes [26] or impedes [27] prompt help-seeking are also reflected in our interviews, which typically reflect real concern from families but describe family responses experienced as both helpful and unhelpful. As in the comparison of carers' and service users' experience of the onset of psychosis by DeHaan and colleagues [15,28], our study found recognition of psychosis and the need to seek help often came sooner among family members and other involved people than for the person experiencing symptoms. Our study helps validate these findings for a diverse, urban UK setting.

The descriptions in our study of responses to early psychotic symptoms help to understand these findings. The vague nature of many early psychotic experiences creates difficulties, especially for those without specialist training, in distinguishing illness symptoms from other motivational or developmental problems. A substantial number of service users (but not carers) remained unconvinced that they had a substantial problem up to the point of contact with mental health services. These problems of recognition of psychosis were sometimes exacerbated by people experiencing psychosis deliberately masking symptoms. GPs in particular were presented with vague, or physical rather than mental, symptoms, illustrating why it is difficult for them to identify early psychosis.

As well as consolidating previous knowledge, this study adds to it in the following ways:

#### The role of community organisations

Engagement of the wider community in identifying and seeking help for psychosis has been advocated for early intervention services recurrently by experts [29] and in government policy [7,30]. To our knowledge, this is the first UK study to focus on the role of non-health organisations in help-seeking for a first episode of psychosis. A number of barriers to workers in community organisations promoting contact with mental health services were identified: these included non-disclosure or active masking of problems by the person experiencing psychosis, failure to recognise problems as psychosis, an insufficiently close relationship or a clearly defined, limited role with the person with psychosis, and preference for a wait and see approach. However, our interviews also revealed that people working in community organisations frequently were willing to intervene and did either seek help directly or encourage the young person or their family to do so. In some instances, community groups also played a valuable role in providing a social identity and support for both service users and their carers throughout the period of developing and recovering from psychosis.

#### Pathways to care

Participants in this study typically reported significant service delays in pathways to care once help-seeking had been initiated. In our sample, these mostly related to the response of GPs and primary care, rather than secondary mental health services.

#### The experience of different ethnic groups

We purposively sampled participants to reflect the ethnic diversity of the local catchment area. Similarities in participants' experiences were more outstanding than differences: we did not find differences between ethnic groups regarding stigma concerns, reservations about mental health services or experiences of service responses. Encouragingly, we found no evidence suggesting discriminatory practice. Our study suggests potential for effective intervention across the whole target population to address the problems of stigma and mistrust of services, rather than clearly indicating tailored initiatives for specific social or ethnic groups.

### Limitations

The limitations of this study relate to sampling issues and the retrospective nature of the data collected. In common with previous studies of people with psychosis [31], lower recruitment of carers than service users was achieved: not all CIEIS service users have involved carers, and reluctance to revisit distressing experiences and time pressures may have impeded carer recruitment. Women from white ethnic groups were over-represented in our small carer sample of nine, reflecting who we were able to recruit. Although small samples are often sufficient to achieve theme saturation in qualitative research [32], a larger sample would increase confidence that our results reflected a full range of carers' experiences.

Our service user sample had similar duration of untreated psychosis to CIEIS service users in general, but deliberately over-represented those in contact with community groups during illness onset, in order to explore the role of these groups in help-seeking. This divergence from the general CIEIS population, and socio-demographic differences between the client base of CIEIS and early intervention services in other parts of the UK may limit the applicability of our findings to a wider first episode psychosis population. Finally, experiences of the onset of illness and first contact with mental health services were reported retrospectively, creating possible lacunae or distortions through recall bias.

### Research Implications

This study describes multiple barriers to prompt treatment for people with first onset psychosis both before help-seeking is initiated and after first contact with services. It supports the conclusion of a recent review [17] that initiatives to reduce DUP should be multi-focused and not (as for instance, GP education campaigns are) limited to reducing service delays. Our study suggests that a very significant component of delay is attributable to difficulties in recognising early symptoms as psychosis and reluctance by young people and their carers to act at the earliest opportunity to signals of a potential threat of psychosis. Help is frequently not sought until a crisis point has been reached. Without a strategy to address these delays, the hope of achieving an optimal reduction in DUP is unlikely to be realised. Further research regarding the most effective means to promote help-seeking for early psychosis is required.

Previous Canadian research has found that the involvement of people working in non-health service community organisations during pathways to care is common [21] but can be associated with treatment delay [22]. Our study suggests some degree of willingness among UK non-health professionals such as teachers, faith group leaders and organisers of social or cultural groups to be involved in helping young people with psychosis or their families to access treatment. However, it also suggests that these people may not always recognise early psychosis, lack knowledge about how to access appropriate services and hold reservations about the benefits of contact with mental health services for young people with whom they work. The crucial question of whether early detection initiatives can overcome these barriers requires further research. There is a need to design interventions targeting community professionals who are in contact with young people during their first onset of psychosis, and to evaluate whether these can improve their effective involvement in help-seeking and play a significant role in reducing DUP.

The interviews in this study reflect the difficulties for all involved in recognising early psychosis. An alternative early detection approach from focusing narrowly on psychosis would be a stepped strategy: first, seeking to improve access to assessment and treatment for distressed young people with a range of mental health problems and associated deterioration in functioning; then second, seeking to improve detection and assessment for early psychosis from within this group. Comparison of the effectiveness and cost effectiveness of broader and more focused strategies to enhance early detection merits investigation.

This study indicates there is probably value in routine monitoring in early intervention services of pathways to care and components of DUP. This could illuminate major contributors to DUP and agencies involved in helping people access treatment, which may vary across cultures and between local service systems and so inform priorities for local initiatives to reduce DUP. The MiDATA Project in the UK [33] provides one means by which this information can be collected.

### Clinical implications

The median DUP for the service user sample in this study was fairly short, coming close to achieving the World Health Organisation 3-month target [34]. Even so, a minority experienced very long DUP and most service users and carers described considerable distress and disruption to their lives following the onset of psychosis. This supports UK government advice that early intervention services should prioritise early detection and education of the wider community about psychosis [7,35].

Interviewees in our study reported not knowing where to get help. Carers also expressed uncertainty about what to do if the person experiencing psychosis was reluctant to accept help. Even in the absence of resources for concerted early detection initiatives, early intervention services may be able to reduce these barriers to accessing treatment through modest changes to service organisation and practice. Feasible and potentially useful actions include: providing direct telephone access to the service; offering advice to concerned family members or involved community professionals, even if a referral cannot be made straight away. With sufficient resource allocation, more concerted campaigns are desirable. Ways to seek prompt referrals for people with first episode psychosis which have been used in large-scale early detection initiatives [36,37] include: establishing regular links between the local early intervention service and GPs and large community organisations, public lectures in schools, colleges or community groups, "open house" events and promotional leaflet distribution. The experience of broader mental health initiatives [38,39] suggests that prominent service user involvement in early detection initiatives, emphasising the stories and experiences of people who live with mental health problems may be an effective means to normalise psychosis and reduce stigma-driven reluctance to contact services. Internet and social networking forums also hold promise as mechanisms through which to reach young people: input from social marketers regarding the most effective media to reach target audiences could usefully inform future initiatives.

## Conclusion

This qualitative study describes the experiences of young people and their carers during the onset of psychosis. It shows how a range of psychosocial factors cause delays in provision of appropriate treatment for young people developing psychosis, despite them and their families experiencing considerable worry. The distress reported by young people and their families, even in relatively brief periods of untreated psychosis, supports prioritisation by early intervention services of promoting swift access to treatment. The study also shows how people's immediate support networks and involved community organisations may either encourage or deter help-seeking. It highlights the need for more research to establish how mental health service initiatives can promote prompt help-seeking for people with first onset psychosis and how not just young people themselves, but also their families and broader social networks including non-health community organisations, can be helped to play a more effective role in this process.

## Abbreviations

DUP: Duration of untreated psychosis; CIEIS: Camden and Islington Early Intervention Service

## Competing interests

The authors declare that they have no competing interests.

## Authors' contributions

SJ, MH, NM, HK, RR and SP contributed to study design. MC and MH conducted interviews with study participants. ST led the analysis of study data with contributions from NM, BLE, MH, MC and SJ. ST, NM, MH, BLE, HK, RR, SP and SJ helped write the paper. All authors read and approved the final manuscript.

## Pre-publication history

The pre-publication history for this paper can be accessed here:

http://www.biomedcentral.com/1471-244X/11/157/prepub

## Supplementary Material

Additional file 1 **"Additional illustrative quotes from qualitative data"**. additional illustrative quotes from the qualitative data Click here for file
