# Supplementary material for: Service user and carer experiences of seeking help for a first episode of psychosis: a UK qualitative study
Source: BMC Psychiatry. 2011 Sep 30;11:157. doi: 10.1186/1471-244X-11-157 (PMC3193166; doi:10.1186/1471-244X-11-157)
Supplement: Additional file 1 — "Additional illustrative quotes from qualitative data". additional illustrative quotes from the qualitative data [file 1471-244X-11-157-S1.DOC]

**“Service user and carer experiences of seeking help for a first episode of psychosis: a UK qualitative study”**

**Tanskanen,S. Morant,N. Hinton,M. Lloyd-Evans,B. Crosby,M. Killaspy,H. Raine,R. Pilling,S. Johnson,S.**

**Additional File 1: Additional Illustrative Quotes from Qualitative Data**

| **Quote number** | **Quote** |
| --- | --- |
| **Section 1: Understandings of symptoms and experiences and associated behavioural responses** | |
| 1.1 | “I suppose it was my pride telling me that there was nothing wrong with me. That these things happen to everybody from all walks of life. And it wasn't until somebody sort of literally shook it out of me, that was my mother-in-law ,that I realised that something really wasn't quite right […]. I was convinced, and it took years until it sunk in that something was wrong.” (Service user; female, 37, Black Caribbean) |
| 1.2 | “Well I’m just beginning to understand all the aspects of the illness because I don’t think I did know enough. And obviously I understand that all his aggression is caused by his paranoia and insecurity so me, err, being such a horrible mother and trying to kick him out of the house and make him, push him to make him go to work or go to school or whatever, did not have positive impact on Tom’s recovery. And the reason was because I was stupid, I didn’t have, I was ignorant about this illness and I didn’t understand that illness...“ (Carer; mother, 55, White Other) |
| 1.3 | “A lot of stuff in my head like war, politics, bad things go into my head. I can’t believe I used to think like that, the drugs. It was horrible, it was evil the things you think. It was the drugs not me.” (Service user; male, 34, White Other) |
| 1.4 | “Yeah I thought to myself, like I said that it was a bad situation that was going to turn around for me. That's another reason why I didn't seek help from the mental health because I didn't want to waste anybody's time really…” (Service user; female, 31, Black Caribbean) |
| 1.5 | “I was stuck up and I was thinking like I can handle it, I can push him straight because if I like stop providing for him he will find some common sense, like find himself a job or perhaps if he works hard physically for one year or two, he will be able to go back to school, get some common sense.” (Carer; mother, 55, White Other) |
| 1.6 | “She [mother] let me just lock myself up in a room and I just stayed there. I wouldn’t come out and eat until it was dark, when it was dark I would come out. But when it was light it was weird man, it was really dodgy, for weeks I must have stayed in all day and then as soon as it come night time I’d come out, close all the curtains, then eat and just do my bit and stay up all night and stay in all day. It was a mess, a real bad mess.” (Service user; male, 28, Mixed Race) |
| 1.7 | “I didn't want to make out that I was having a breakdown like.” (Service user: male, 26, Asian Bangladeshi) |
| **Section 2: Help seeking processes** | |
| 2.1 | “I wasn't really aware of what was happening until I was contacted by the [CI]EIS.” (Service user; female, 25, Mixed Race) |
| 2.2 | “[…] it was quite sudden. Although he said he had had it for a few weeks maybe even longer but we hadn’t noticed anything. I think it came on quite dramatically because he was actually quite scared and frightened. I was having to walk the streets with him and be with him all the time really. The first thing I did was made an appointment to see the GP and take him along to the GP.” (Carer; mother, 59, White British) |
| 2.3 | “It took me quite a long time to understand that there was a mental issue there but it wasn’t until she started to become very ill. She actually had a crisis.” (Carer; mother-in-law, 68, White British) |
| **Section 3: Beliefs and knowledge regarding mental health services** | |
| 3.1 | “I was a bit embarrassed and a bit afraid. And I thought, not only did I think everybody else could hear the voice anyway... if they didn't, I didn't want them to know. 'Cause it's kind of a stigma, isn't it, being labeled, hearing voices…[…] And with kids, you know, you don't want to have a mental illness with children, 'cause the social services are gonna get involved, which they have tried to, and make you feel that they can take your kids away. And that's the last thing I want, you know what I mean, so I'd rather people didn't know.” (Service user; female, 27, Black Caribbean) |
| 3.2 | “I didn’t want my daughter associated with other psychiatric patients. I didn’t want her to think what she had was psychiatric.” (Carer; mother, 58, White Other) |
| 3.3 | “Yeah I wasn't um when I was going through my brief turmoil I wasn't sure who to go to or who to speak to, who I would be able to trust or um who would give me the right information really.” (Service user; female, 31, Black Caribbean) |
| 3.4 | “I have a feeling I did try. I mean I spoke to friends about it. I didn’t even think to go to a psychiatrist, I wouldn’t even have known where to go. I mean I didn’t know this place [CIEIS] here existed. Had I known about it and had they had a helpline or something, I might have done that and I think that would be very, very good if they had a helpline for confused and worried parents or parents-in-law or friends.” (Carer; mother-in-law, 68, White British) |
| **Section 4: Responses of social networks to illness onset and help-seeking** | |
| 4.1 | “I didn't actually go to my GP, I went to my husband and my mother-in-law first and they said "Oh don't worry about it, it could be just a phase you are going through". But it wasn't really a phase it was just the first symptoms that something was wrong.” (Service user; female, 37, Black Caribbean) |
| 4.2 | “No-one [at college] had ever spoken to me about my problem with behaviour or said ’are you okay, or you seem to be under a lot of pressure, or having some..’. No- one has ever asked me ‘do you suffer with mental health?’ or anything like that.” (Service user; female, 25, Mixed Race) |
| 4.3 | “The rest of the congregation when they used to come and visit me or the ones I was studying the Bible with together. They came to see me, they knew I wasn't well, but I never asked for their help directly so they felt like they didn't want to invade me so they didn't address me to anywhere.” (Service user; male, 27, White Other) |
| 4.4 | “I just didn't feel comfortable with telling them [tutors] like I've got these problems.  Because lecturers for me are much more further apart as regards a relationship.  So I would rather be seen by someone who is in the profession because they know what to expect.” (Service user; male, 21, White Other) |
| **Section 5: Professional responses and service-related delays** | |
| 5.1 | “He [GP] didn’t make the referral. Yeah, he didn’t do anything. […] ‘Just here’s them [tablets], take them and they will help you and calm you down a bit.’ They didn’t do nothing, just made me worse, I was erratic, very erratic.” (Service user; male, 28, Mixed Race) |
| 5.2 | “Well I was worried sick. I had taken him to the doctor as … as he actually did become really unwell and was coming towards that point. I took him to the Counselling Centre which is supposedly self referral. Yes the self referral to go through the door then when you ask for help you have to go to the GP. So you know.. I hadn’t been getting help. The help I had been asking for the help from the school, the GP I hadn’t been getting the help you know two, three times, four times.” (Carer; mother, 60, White British) |
| 5.3 | “He was using very abusive words towards me which broke my heart. On one occasion we had disagreement […] and he pushed me on the floor. That was the kind of point that I decided well I cannot accept this kind of behaviour anymore and I called the police. When the police came they said is there something strange going with his behaviour and before anything else I would like him to be consulted by a psychiatrist, which he was. The psychiatrist said there was nothing really wrong with him apart from his drug abuse” (Carer; mother, 55, White Other) |
| 5.4 | “When we got there [GP surgery] he started making out as though he had gone for a medical problem and that he had a lump somewhere. […] No, and even now he’s not acknowledged that he’s hearing any voices. He never admits to it.” (Carer; mother, 59, White British) |
